# Uncovering Evolutionary Adaptations in Common Warthogs through Genomic Analyses

**DOI:** 10.3390/genes15020166

**Published:** 2024-01-27

**Authors:** Xintong Yang, Xingzheng Li, Qi Bao, Zhen Wang, Sang He, Xiaolu Qu, Yueting Tang, Bangmin Song, Jieping Huang, Guoqiang Yi

**Affiliations:** 1 Shenzhen Branch, Guangdong Laboratory of Lingnan Modern Agriculture, Key Laboratory of Livestock and Poultry Multi-Omics of MARA, Agricultural Genomics Institute at Shenzhen, Chinese Academy of Agricultural Sciences, Shenzhen 518124, China; tty970512@126.com (X.Y.); lxz2019@alu.cau.edu.cn (X.L.); baoqinetwon@163.com (Q.B.); wangzhenchn_zz@163.com (Z.W.); hesang@caas.cn (S.H.); qquuxxiiaaoolluu@163.com (X.Q.); 17689274543@163.com (Y.T.); bangminsong@163.com (B.S.); 2 State Key Laboratory for Conservation and Utilization of Subtropical Agro-Bioresources, Guangxi University, Nanning 530005, China; huangjieping1989@126.com; 3 School of Life Sciences, Henan University, Kaifeng 475004, China; 4 Kunpeng Institute of Modern Agriculture at Foshan, Agricultural Genomics Institute at Shenzhen, Chinese Academy of Agricultural Sciences, Foshan 528226, China; 5 Bama Yao Autonomous County Rural Revitalization Research Institute, Bama 547500, China

**Keywords:** warthog, gene family, phylogenetic relationships, divergence, phenotype, QTL

## Abstract

In the Suidae family, warthogs show significant survival adaptability and trait specificity. This study offers a comparative genomic analysis between the warthog and other Suidae species, including the Luchuan pig, Duroc pig, and Red River hog. By integrating the four genomes with sequences from the other four species, we identified 8868 single-copy orthologous genes. Based on 8868 orthologous protein sequences, phylogenetic assessments highlighted divergence timelines and unique evolutionary branches within suid species. Warthogs exist on different evolutionary branches compared to DRCs and LCs, with a divergence time preceding that of DRC and LC. Contraction and expansion analyses of warthog gene families have been conducted to elucidate the mechanisms of their evolutionary adaptations. Using GO, KEGG, and MGI databases, warthogs showed a preference for expansion in sensory genes and contraction in metabolic genes, underscoring phenotypic diversity and adaptive evolution direction. Associating genes with the QTLdb-pigSS11 database revealed links between gene families and immunity traits. The overlap of olfactory genes in immune-related QTL regions highlighted their importance in evolutionary adaptations. This work highlights the unique evolutionary strategies and adaptive mechanisms of warthogs, guiding future research into the distinct adaptability and disease resistance in pigs, particularly focusing on traits such as resistance to African Swine Fever Virus.

## 1. Introduction

Suidae are widely distributed around the world [1]. The warthog, a member of the Suidae, is native to the African continent, and its natural habitat has always been in the wild [2]. The African landscape, characterized by its varied topographical and climatic regions, has resulted in the emergence of distinct ecological niches, each fostering the evolution of unique species and subspecies [3]. Such ecological specialization can be evidenced by how species respond to and interact with their habitats [4,5,6]. These interactions catalyze biodiversity variations and spur adaptive evolutionary shifts, molding the evolutionary lineage and phenotypic characteristics of organisms through the ages [7]. A notable example of such evolutionary differentiation can be observed between the river buffalo and the swamp buffalo [8]. Both, despite sharing a common ancestry, have manifested distinct genotypic and phenotypic adaptations to their specific environments [9,10]. Among the Suidae family, a particularly notable distinction is that the warthog can carry the African swine fever virus (ASFV) without displaying any clinical symptoms. In stark contrast, ASFV proves fatal to *Sus scrofa* [11,12,13,14]. This distinction potentially underscores the notable evolutionary and adaptive variations within the Suidae family.

Biological evolution is underpinned by a nuanced interrelationship between genotypes (an organism’s complete set of genetic material) and phenotypes (its observable traits). Genotypic variations arise from factors like mutations, genetic recombination, and gene flow [15,16,17,18]. Over time, these genetic alterations can accumulate, resulting in notable differences even among individuals of the same population [19,20]. Taking the Suidae family as an example, the warthog and the Eurasian pig, despite their shared lineage, display marked differences. They have distinct chromosomal counts and show reproductive isolation [21]. In terms of physical characteristics, variations in body size, skin tissue, and other traits are evident between the two. Yet, intriguingly, they maintain a substantial genomic homology [22,23]. There are various reasons behind this phenomenon, with the expansion and contraction of gene families being one of the primary contributors.

A gene family refers to a group of genes derived from a common ancestral gene, typically similar in structure and function [24]. Gene family expansion, facilitated by mechanisms like tandem duplications or transposable element-mediated duplications, leads to an increase in gene count, potentially introducing new gene functions [25]. For instance, the expansion of *HOX* gene families in vertebrates has contributed to the complexity of their body plans and allowed for greater diversification of structures [26,27]. Conversely, gene family contraction can be achieved through gene loss or the formation of pseudogenes. Gene loss may result from gene non-functionalization or complete deletion. Pseudogenes are a specific type of gene that loses its original function but remains in the genome [28,29]. A classic example of this is the olfactory receptor (OR) gene family in humans, where a significant portion have become pseudogenes, reflecting our reduced reliance on smell as compared to other mammals [30,31,32,33]. While expansion provides the raw material for adapting to environmental changes or developing new biological functions, contraction can help eliminate non-essential or detrimental genes, thereby enhancing an organism’s adaptability and survival. In essence, these factors collectively drive the intricate process of biological evolution.

In most of these studies, the primary focus has been on whether the nature of gene family contraction and expansion is driven by strong artificial or natural selection. However, there has been limited attention to the implications of such findings on quantitative and qualitative phenotypic traits of the species itself. Consequently, our study integrates the impact of the expansion and contraction of gene families on the phenotypic traits of the species, offering a comprehensive insight into the adaptive evolution of warthogs. Importantly, this integrative research approach may enrich and benefit future perspectives and angles in studying the pathogenicity of warthogs.

## 2. Materials and Methods

### 2.1. Identification of Single-Copy Orthologous Genes and Gene Families

We extracted protein sequences from the genomes of four Suidae species (Duroc pig, Red River hog [22], Luchuan pig [34], and Common warthog) and four non-Suidae animals (cow, sheep, sperm whale, and horse) based on the longest transcript of each gene and removed sequences shorter than 30 amino acids in length. Whales, cows, and pigs belong to the same evolutionary lineage within the Artiodactyla order [35,36]. Horses, as Perissodactyla animals, serve as an appropriate outgroup for constructing evolutionary relationships [37]. Selecting these animals ensures the accuracy of our phylogenetic relationship analysis. OrthoFinder (v.2.5.4) [38,39,40] was utilized to identify orthologous genes and gene families among the input datasets. From the OrthoFinder analysis, 8868 single-copy orthologous genes were identified across all 8 species, ensuring that each gene is represented only once in each genome.

### 2.2. Phylogenetic Analysis with RaxML

The protein sequences of the 8868 single-copy orthologous genes were aligned using MUSCLE (v.3.8.1) [41]. And we used the default parameters of trimAl (v.1.2.1) [42] to trim away the non-conservative portions. The RAxML [43] was used for phylogenetic tree construction employing the amino acid model (PROTGAMMAJTT). The JTT (Jones–Taylor–Thornton) matrix is a well-established amino acid substitution model that has been widely used due to its suitability for a broad range of datasets [44,45,46]. RAxML is optimized to work efficiently with the PROTGAMMAJTT model, ensuring fast and reliable tree inference, which was particularly important given the large size of our dataset.

### 2.3. Divergence Time

The protein sequences initially aligned with MUSCLE were translated back to concatenated coding sequences (CDSs) using ParaAT [47]. This ensures that the resulting nucleotide alignments are in-frame and reflect the amino acid homology accurately. The accurate back-translation of aligned protein sequences is critical for subsequent phylogenetic analyses, as it preserves the codon integrity and evolutionary signals contained within the coding sequences. We used the CDSs of 8868 single-copy orthologous genes to estimate the divergence times for the eight species in our analysis based on the species tree. This estimation was carried out using MCMCTree, part of the PAML (v.4.9j) [48]. For calibration, we used fossil evidence described in previous studies. The analysis included two fossil constraints: (i) 47–58 Ma is the divergence time between sperm whales and cattle based on previous studies, and (ii) less than 15 Ma is the divergence time between cattle and sheep according to prior research [22,36].

### 2.4. Expansion and Contraction of Gene Families

We compared the cluster sizes of gene families from each of the eight sequenced genomes with their corresponding ancestral gene families using the Café [49] program, which operates on a probabilistic graphical modeling approach. Utilizing CAFE (v5.0), gene family size transition rates from parent to child nodes can be calculated, aiding in ancestral species gene family size inference. Concurrently, conditional likelihoods serve as the statistical metric for deriving *p*-values for each distinct lineage [50]. Gene families with a *p*-value < 0.05 were identified as having undergone significant expansion and contraction within the Suidae clade.

### 2.5. GO and KEGG Analysis

We utilized the g:Profiler. https://biit.cs.ut.ee/gprofiler/ (accessed on 12 October 2023) [51]. We adopted the mouse genome as a reference due to its well-annotated genomic resources. First, our target genes were mapped to their mouse orthologs using g:Profiler-g:Convert. With this set of mouse orthologous genes, we conducted the GO and KEGG enrichment analyses. Enriched GO terms and KEGG pathways were identified based on a significance threshold of *p*-value (*p* < 0.05).

### 2.6. Phenotype and QTL Analysis

We mapped the target genes to annotation file (*Sus scrofa* 11.1) to obtain gene positions. The corresponding genes were then converted to mouse orthologous genes and phenotype analysis was conducted in the MGI database. https://www.informatics.jax.org/ (accessed on 18 October 2023) [52]. Using the regions of all genes as the background set, we conducted enrichment analysis with the QTLdb-pigSS11 (Animal QTL Database). https://www.animalgenome.org/cgi-bin/QTLdb/ (accessed on 3 November 2022) [53].

## 3. Results

### 3.1. Identification and Comparison of Homologous Genes and Gene Family

**Figure 1:**
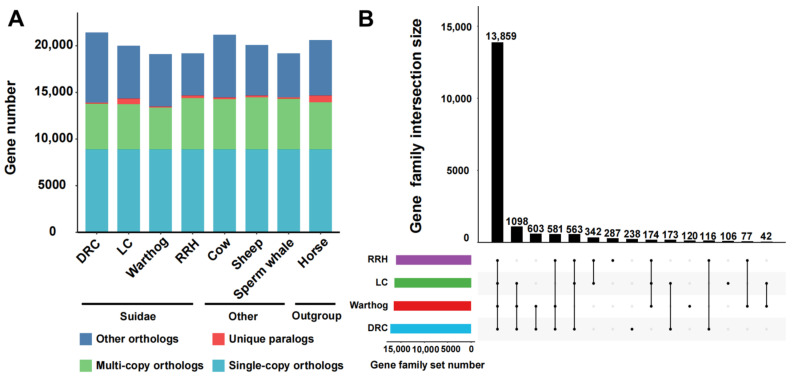
Comparative analysis of homologous genes and gene families. (**A**) Homologous gene quantity and types between Suidae species (Duroc pig (DRC), Luchuan pig (LC), Common warthog (warthog), and Red river hog (RRH)) and other species (cow, sheep, sperm whale, and horse). (**B**) Shared and unique gene families in Suidae species. Lines with black dots signify shared relationships. Black dots without lines indicate uniqueness.

We selected the Luchuan pig [34] and Duroc pig, representative pig breeds from China and the West, for comparison with the warthog. Additionally, we incorporated the Red River hog. Geographically, the warthog’s distribution is proximate to that of the Red River hog [22,54]. For a robust evolutionary analysis, we included non-swine species with well-established evolutionary relationships (Table 1). From these eight genomes, we pinpointed 8868 single-copy orthologous genes (Figure 1A). Subsequently, we compared the number of different types of orthologous genes. The unique gene count in warthogs does not significantly differ from other suid species, but the quantity of multi-copy orthologous genes is lower than in other suid species. The RRH has more unique genes and multi-copy orthologous genes than the warthog (Figure 1A). For Suidae animals, we assigned genes into gene families, classifying them as common (shared across all species), partially shared, or species-specific. During the statistical process, we do not exclude the presence of single copies in one or several breeds. The RRH has more unique gene family’s numbers than the warthog, but there are also gene family’s numbers shared only by the warthog and RRH (Figure 1B). Multi-copy forms are instrumental in organismal evolution and adaptability, serving as a foundational mechanism for gene family creation. These findings highlight that despite the close geographical proximity and shared African wild species status of both the warthogs and RRHs, they exhibit distinct genomic evolutionary patterns.

### 3.2. Phylogenetic Relationships and Divergence Time

**Figure 2:**
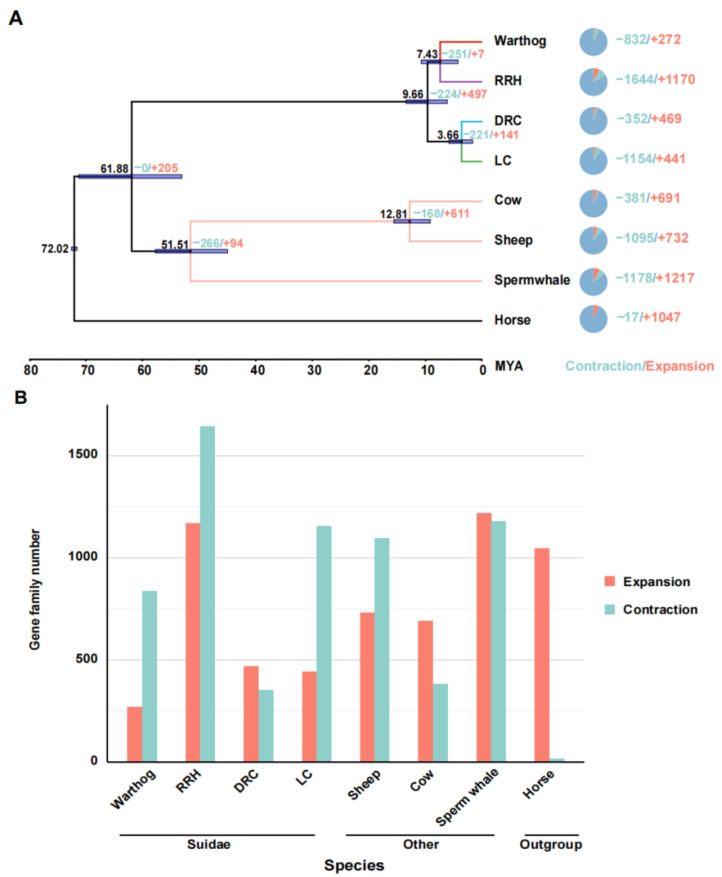
Phylogenetic relationships, divergence time analysis, and expansion and contraction of gene families. (**A**) Estimation of species phylogeny, divergence time, and changes in gene family sizes (contraction and expansion). Horses are not assigned a distinct branch color. Species with a well-established evolutionary relationship share consistent branch colors. The four distinct Suidae species are differentiated by unique colors. Horizontal bars at the nodes represent the 95% HPD confidence intervals. The left node side shows divergence time; the right side details gene family changes. The pie chart on the far right illustrates the proportional representation of gene families that have contracted or expanded. The unit for divergence time is million years. (**B**) Trends in gene family contraction and expansion.

To clarify the phylogenetic relationships and divergence times among Suidae species, we analyzed 8868 single-copy orthologous protein sequences, using the horse as the outgroup [55]. The phylogenetic tree was constructed using protein sequences with an amino acid model. The phylogenetic tree indicates a distinct common ancestral branch for the warthogs and Red River hogs, while the Duroc and Luchuan pigs belong to another branch, consistent with previous research [22,56]. Subsequently, we translated the protein sequences of single-copy orthologous genes into their corresponding concatenated coding sequences (CDSs). Additionally, we utilized the fossil records of non-Suidae animals with well-established evolutionary relationships as calibration points (sperm whale; cow; sheep) [36,57]. The results indicate that there was a divergence time of approximately 9.7 million years ago (95% highest posterior density, HPD = 13.9–6.2) between African suids and Eurasian pigs, and the divergence between warthogs and RRHs occurred approximately 7.43 million years ago, (95% highest posterior density, HPD = 10.8–4.3) with the Eurasian species diverging late (Figure 2A). Warthog and RRH began to diverge earlier than the Eurasian species, suggesting they possess unique evolutionary factors and patterns compared to Eurasian pigs.

### 3.3. Expansion and Contraction of Gene Families

**Figure 3:**
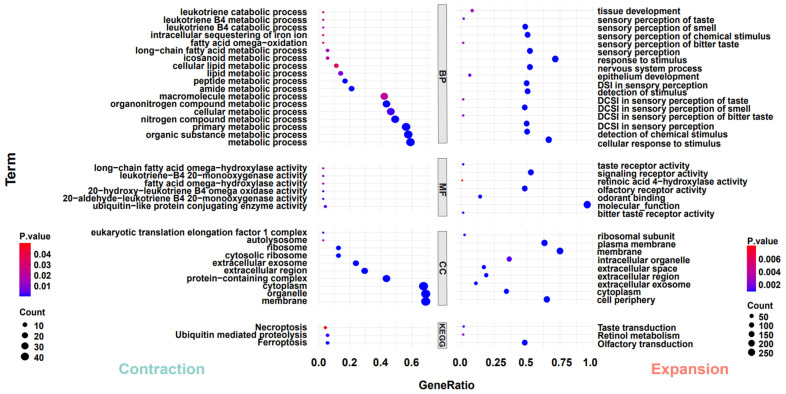
Analysis of GO and KEGG pathways. The left side analyzes genes in significantly contracted (*p* < 0.05) gene families in warthog, while the right side focuses on those in significantly expanded (*p* < 0.05) gene families in warthog. Functional enrichment classification: biological process (BP), molecular function (MF), cellular component (CC).

To investigate the adaptive evolution of warthogs during their evolutionary history, we analyzed the contraction and expansion of gene families across all animals in the phylogenetic tree, utilizing data derived from the allocation of genes to their respective families and insights from multicopy genes. In light of the inherent preferences of our analysis tools, we deemed it appropriate to redefine gene families based on the output results. One potential advantage of this approach is it considers genes that may have undergone sequence modifications throughout evolution, potentially influencing their homology [40]. The results indicate that throughout the evolutionary process, all species exhibit varying degrees of contraction and expansion in gene families, including those within the Suidae family (Figure 2A). However, the trends in gene family contraction and expansion are inconsistent within the Suidae family. In their evolutionary progression, Duroc pigs have more gene families that have expanded than contracted, while Warthogs, Red River Hogs, and Luchuan pigs have the opposite trend (Figure 2B). This may be due to the DRC being subjected to more intense artificial selection. In previous studies, a similar trend was also observed [56]. Subsequently, we converted the genes from the prominently expanded and contracted warthog gene families into their human homologues and performed Gene Ontology and Kyoto Encyclopedia of Genes and Genomes (GO and KEGG) analyses on these gene families (Figure 3). Significantly expanded gene families in warthogs are primarily associated with sensory stimuli, including taste, smell, and vision. (e.g., GO:0004984~ olfactory receptor activity, GO:0007608~ sensory perception of smell, KEGG:00830~ retinol metabolism.) Among them, the olfactory function is notably enriched in the classic olfactory receptor (OR) gene family [58,59]. Originating from the *UGT* gene family, the *UGT2A3* and *UGT2A1* genes are notably associated with retinol metabolism. In human studies, the *UGT* gene family is vital for maintaining homeostasis by facilitating the elimination of potentially harmful compounds [60]. On the other hand, genes like *TAS2R16*, *TAS2R7*, *TAS2R42*, *TAS2R9*, and *TAS2R10* are significantly associated with taste. In various species, the *TAS2R* bitter taste receptor gene activates selectively based on specific environmental conditions [61,62] The prominently contracted gene families in warthogs are chiefly associated with metabolic processes. We infer that warthogs, as wild species, exhibit pronounced sensitivity to environmental stimuli. This heightened sensitivity may have driven the evolution of their acute sensory perceptions, enhancing their capabilities in foraging and predator evasion. This further substantiates the adaptive evolution of warthog pig.

### 3.4. Gene Family Contraction and Adaptive Avoidance

**Figure 4:**
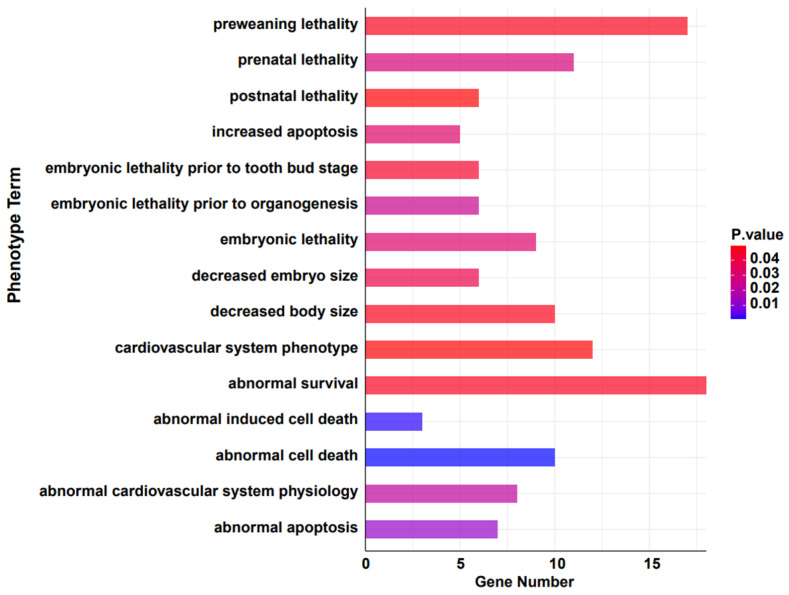
Phenotypic analysis of significantly contracted (*p* < 0.05) gene families in warthog.

To further investigate if the contraction of gene families is associated with avoiding detrimental evolutionary effects, we converted the remaining genes from these families to their murine orthologs and examined their phenotypic associations using the MGI database [52]. The results suggest that the significantly contracted genes are notably correlated with survival rates under varying conditions (Gene Count number top 20, *p* < 0.05) (Figure 4 and Table 2). *Cul3* and *Cycs* are associated with several conditions including abnormal survival, preweaning lethality, prenatal lethality, embryonic lethality prior to organogenesis, and decreased embryo size. This indicates a crucial role in embryonic development and survival. Other studies have shown that it significantly affects tumors and cellular apoptosis [63,64,65]. Genes such as *Eef1a1* [66], *Eef1a2* [67], and *Fth1* consistently appear in contexts related to survival, cell death, and cardiovascular system phenotypes, highlighting their potential roles in key physiological processes [68]. *Krt1* [69] and *Lama2* [70] show a breadth of associations, from abnormal survival to cardiovascular system phenotype and decreased body size, emphasizing their diverse functional implications. *Myh3*, *Myh7*, and *Nrxn2* are tied to various survival and developmental phenotypes, suggesting a pivotal role in early life stages. Moreover, earlier studies have indicated that the *Myh3* gene exhibits a strong pathogenicity in Suidae (pig-related) animals, and it is associated with abnormalities in skeletal and muscular development [71]. *Ntrk3*, *Nup153*, and *Pom121* [72,73,74] present themselves across several survival-associated categories, emphasizing their potential importance in life preservation. These results correspond to the functional enrichment analysis of the contracted gene families. This suggests that, over the course of long-term evolution in the wild, the warthog has, through certain patterns, attenuated and reduced gene families unfavorable for its survival. It hints at a unique adaptive evolutionary strategy that the warthog may have developed to circumvent unfavorable factors, though this warrants further investigation and discussion.

### 3.5. Association between Expanded and Contracted Gene Families with QTLs

**Figure 5:**
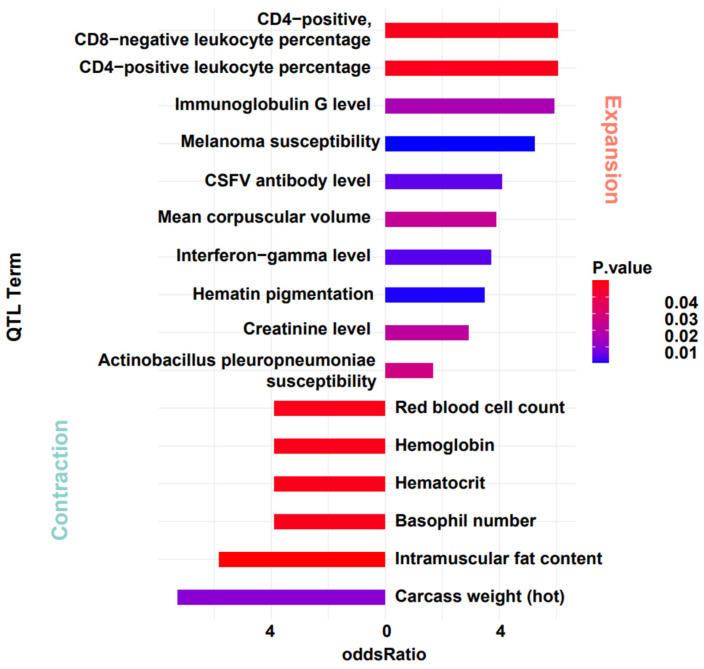
QTL Analysis of gene families with significant expansion and contraction in warthog.

For the notably contracted and expanded gene families, we further investigated their association with quantitative trait loci (QTL). Given the conservation of gene function, we mapped genes from the warthog’s significant expanded and contracted gene families onto DRC annotation files. After obtaining the gene localization data, we designated the contracted and expanded gene regions as the target sets. Using the regions of all genes as the background set, we conducted enrichment analysis with the QTLdb-pigSS11 database [53]. The results show that these gene locations are significantly enriched in QTL entries related to cell levels and Classical Swine Fever Virus (CSFV). Genes from the contracted gene family are enriched in entries related to blood cells (e.g., red blood cell count, hematocrit, hemoglobin). Genes from the expanded gene family are enriched in entries related to immunity (e.g., CSFV antibody level, immunoglobulin G level, creatinine level, CD4-positive, CD8-negative leukocyte percentage, CD4-positive leukocyte percentage). After mapping these entries back to their specific gene names, we obtained an intriguing result. Genes related to olfaction that expanded significantly during evolution are located in immune-related QTL regions, and some genes exhibit pleiotropic effects (Figure 5 and Table 3). Although these findings may be influenced by the extended regions of some QTLs and the regulatory effects of numerous genes, it prompted new reflections on evolutionary changes. The impact mechanism of evolutionary changes on organisms may not be solely focused on functionality. There is a potential for a domino effect, and future research aims to delve deeper into the specific causes of this phenomenon.

## 4. Discussion

In this work, we utilized the genomic data from species including the Common warthog, Red River hog, Duroc pig, Luchuan pig, cow, sheep, sperm whale, and horse as a foundation for our comparative genomic analysis. From these data, we discerned 8868 single-copy orthologous genes. Subsequently, we examined the shared and unique gene families prevalent among the Suidae species. At the core of our findings is the intricate balance of gene expansion and contraction, which seems to be intertwined with a species’ survival and adaptation mechanisms. A salient observation from our analyses is the distinct evolutionary trajectories of warthogs and RRHs, despite their geographical proximities. This suggests that even in analogous environments, species might adopt disparate genomic strategies to ensure survival and reproductive success [75]. The phylogenetic analysis further emphasizes the unique evolutionary dynamics, as evidenced by the estimated divergence times between the species. The use of protein sequences in delineating the common ancestral lineage offers robust insights into the genomic history of these species [76,77]. The role of gene family expansion and contraction in adaptation becomes evident [29]. For instance, sensory genes related to olfaction and taste, including the OR and *TASR2* gene families, have experienced notable expansion [58,59,61,62]. This suggests an evolutionary emphasis on heightened environmental sensitivity, likely facilitating efficient foraging and predator evasion in wild settings [78]. Conversely, the contraction observed in certain gene families highlights another aspect of the adaptive strategy. Genes such as *Myh3*, *Cul3*, *Cycs*, *Eef1a1*, and *Eef1a2* play direct roles in survival and developmental processes [63,64,65,66,67,71]. Their contraction in warthogs could be a strategic move, minimizing or eliminating certain genetic elements that might be unfavorable for survival in their specific environment [79]. Furthermore, the association between these contracted gene families and phenotypic outcomes, particularly survival rates, reveals a deeper layer of genomic regulation. The involvement of genes such as *Krt1*, *Lama2*, *Ntrk3*, and *Nup153* in various survival and developmental phenotypes indicates a broad genetic framework governing these processes [69,70,72,73,74]. These findings, when combined with the functional enrichment analysis, elucidate an evolutionary narrative where gene families might be selectively downsized to streamline the warthog’s survival mechanisms.

Our study also delves into the nexus between the changed gene families and QTLs. The potential overlap between genes and QTLs, especially those related to olfaction, and immune-related QTL regions, highlights the possibility of pleiotropic effects. Such effects suggest an evolutionary ‘domino effect’, where alterations in one genetic pathway might influence others, emphasizing the interwoven nature of genomic evolution [80,81,82].

## 5. Conclusions

In summary, our study underscores the complex interplay of gene family dynamics in adaptive evolution, spotlighting genes that potentially play pivotal roles in survival and environmental adaptation. Expansion provides the raw material for adapting to environmental changes or developing new biological functions. In contrast, contraction helps eliminate non-essential or detrimental genes, enhancing an organism’s adaptability and survival capabilities. Fundamentally, these factors collectively propel the intricate evolutionary journey of the warthog. Although we have clarified that warthogs have shown positive effects in aspects such as olfaction, hearing, immunity, and resistance to African Swine Fever during their adaptive evolutionary process, it is evident that the expansive field of evolutionary genetics still harbors many unresolved mysteries, inviting deeper investigations. In subsequent studies, to decipher genetic mechanisms driving the evolution of Suidae animals and traits of economic importance in domestic pigs, particularly disease resistance for the African Swine Fever virus, more extensive datasets and bioinformatics analyses, like case-control GWAS, selective sweep scanning, gene expression analysis, and single-cell omics, should be included. Additionally, employing CRISPR experiments for functional validation will be essential in advancing our understanding in this critical area.

## Figures and Tables

**Table 1:** Selected species and genomic sources.

Genome Version	Genome Source	Species	Scientific Name
ROS_Pafr_v1	NCBI	Common warthog	*Phacochoerus africanus*
River_1.0	CNCB	Red river hog	*Potamochoerus porcus*
Sscrofa11.1	Ensembl	Duroc pig	*Sus scrofa domesticus*
CNP0001159	CNSA	Luchuan pig	*Sus scrofa domesticus*
ARS-UCD1.2	Ensembl	Cow	*Bos taurus*
Oar_rambouillet_v1.0	Ensembl	Sheep	*Ovis aries*
ASM283717v2	Ensembl	Sperm whale	*Physeter macrocephalus*
EquCab3.0	Ensembl	Horse	*Equus ferus caballus*

**Table 2:** Phenotype term and genes.

Phenotype Term	Gene Count
MP:0010769-abnormal survival	*Cul3*, *Cycs*, *Eef1a1*, *Eef1a2*, *Fth1*, *Krt1*, *Lama2*, *Myh3*, *Myh7*, *Nrxn2*, *Ntrk3*, *Nup153*, *Pom121*, *Rps2*, *Sox3*, *Tpt1*, *Ube2n*, *Ybx1*
MP:0010770-preweaning lethality	*Cul3*, *Cycs*, *Eef1a1*, *Fth1*, *Krt1*, *Lama2*, *Myh3*, *Myh7*, *Nrxn2*, *Ntrk3*, *Nup153*, *Pom121*, *Rps2*, *Sox3*, *Tpt1*, *Ube2n*, *Ybx1*
MP:0005385-cardiovascular system phenotype	*Ap1ar*, *Cmtm3*, *Cul3*, *Fth1*, *Krt1*, *Mlip*, *Myh7*, *Myh7b*, *Nrxn2*, *Ntrk3*, *Nup153*, *Ybx1*
MP:0000313-abnormal cell death	*Cul3*, *Cycs*, *Eef1a2*, *Fth1*, *Hnrnpk*, *Lama2*, *Myh3*, *Tpt1*, *Ube2n*, *Vdac2*
MP:0002080-prenatal lethality	*Cul3*, *Cycs*, *Eef1a1*, *Fth1*, *Myh3*, *Myh7*, *Nup153*, *Pom121*, *Tpt1*, *Ube2n*, *Ybx1*
MP:0001265-decreased body size	*Cul3*, *Eef1a2*, *Hnrnpk*, *Krt1*, *Lama2*, *Myh3*, *Nrxn2*, *Ntrk3*, *Rps2*, *Sox3*
MP:0001544-abnormal cardiovascular system physiology	*Cmtm3*, *Cul3*, *Fth1*, *Krt1*, *Mlip*, *Myh7*, *Ntrk3*, *Ybx1*
MP:0008762-embryonic lethality	*Cul3*, *Cycs*, *Fth1*, *Myh7*, *Nup153*, *Pom121*, *Tpt1*, *Ube2n*, *Ybx1*
MP:0001648-abnormal apoptosis	*Cul3*, *Cycs*, *Hnrnpk*, *Myh3*, *Tpt1*, *Ube2n*, *Vdac2*
MP:0013292-embryonic lethality prior to organogenesis	*Cul3*, *Fth1*, *Nup153*, *Pom121*, *Tpt1*, *Ube2n*
MP:0006042-increased apoptosis	*Cul3*, *Hnrnpk*, *Myh3*, *Tpt1*, *Ube2n*
MP:0013293-embryonic lethality prior to tooth bud stage	*Cul3*, *Fth1*, *Nup153*, *Pom121*, *Tpt1*, *Ube2n*
MP:0002082-postnatal lethality	*Eef1a1*, *Krt1*, *Lama2*, *Nrxn2*, *Ntrk3*, *Sox3*
MP:0008942-abnormal induced cell death	*Eef1a2*, *Fth1*, *Tpt1*
MP:0001698-decreased embryo size	*Cul3*, *Cycs*, *Tpt1*, *Ybx1*

**Table 3:** Positions of olfactory-related genes and QTLs in the expanded gene family in warthog.

Chr	Start	End	Gene	Chr	Start	End	QTL
2	56,631,761	56,632,714	*OR2G2*	2	27,258,744	64,868,911	Melanoma susceptibility QTL (7596)
2	54,835,060	54,835,995	*OR2M4*	2	27,258,744	64,868,911	
2	53,163,848	53,164,789	*OR2T27*	2	27,258,744	64,868,911	
2	53,186,402	53,187,358	*OR2T1*	2	27,258,744	64,868,911	
2	53,209,020	53,209,946	*OR2T6*	2	27,258,744	64,868,911	
2	56,615,141	56,616,070	*OR2G3*	2	27,258,744	64,868,911	
4	91,015,547	91,016,509	*OR10J1*	4	85,941,434	100,831,287	Hematin pigmentation QTL (4900)
4	91,788,359	91,796,215	*OR10R2*	4	85,941,434	100,831,287	
4	91,024,840	91,025,775	*OR10J4*	4	85,941,434	100,831,287	
2	12,603,912	12,604,838	*OR5B21*	2	11,271,548	35,884,929	Interferon-γ level QTL (12,334)
2	12,707,442	12,708,380	*OR5B3*	2	11,271,548	35,884,929	
2	11,964,446	11,965,381	*OR4D11*	2	11,271,548	35,884,929	
2	11,972,196	11,973,130	*OR4D10*	2	11,271,548	35,884,929	
2	11,783,066	11,784,022	*OR10V1*	2	11,271,548	35,884,929	
2	14,510,944	14,511,873	*OR4B1*	2	11,271,548	35,884,929	
9	50,634,384	50,635,262	*OR6M1*	9	36,028,377	51,399,180	CSFV antibody level QTL (37,543)
9	50,613,020	50,613,958	*OR6X1*	9	36,028,377	51,399,180	
9	51,126,795	51,127,733	*OR10D3*	9	36,028,377	51,399,180	
9	50,758,169	50,759,113	*OR4D5*	9	36,028,377	51,399,180	
9	50,924,623	50,925,621	*OR10G6*	9	36,028,377	51,399,180	
12	49,103,075	49,104,016	*OR1D5*	12	45,861,635	52,179,865	Mean corpuscular volume QTL (12,302)
12	49,379,227	49,380,174	*OR3A2*	12	45,861,635	52,179,865	
12	49,389,731	49,390,818	*OR3A1*	12	45,861,635	52,179,865	
2	12,603,912	12,604,838	*OR5B21*	2	11,501,580	150,670,698	Actinobacillus pleuropneumoniae susceptibility QTL (37,557)
2	56,631,761	56,632,714	*OR2G2*	2	11,501,580	150,670,698	
2	54,835,060	54,835,995	*OR2M4*	2	11,501,580	150,670,698	
2	53,163,848	53,164,789	*OR2T27*	2	11,501,580	150,670,698	
2	53,186,402	53,187,358	*OR2T1*	2	11,501,580	150,670,698	
2	53,209,020	53,209,946	*OR2T6*	2	11,501,580	150,670,698	
2	56,615,141	56,616,070	*OR2G3*	2	11,501,580	150,670,698	
2	12,707,442	12,708,380	*OR5B3*	2	11,501,580	150,670,698	
2	11,964,446	11,965,381	*OR4D11*	2	11,501,580	150,670,698	
2	11,972,196	11,973,130	*OR4D10*	2	11,501,580	150,670,698	
2	11,783,066	11,784,022	*OR10V1*	2	11,501,580	150,670,698	
2	14,510,944	14,511,873	*OR4B1*	2	11,501,580	150,670,698	

## Data Availability

The genomic datasets analyzed for this study are sourced from recognized public repositories. Specifically, sequence data and related genomic information were retrieved from the National Center for Biotechnology Information (NCBI), Ensembl Genome Browser, China National Center for Bioinformation (CNCB), and China National GeneBank DataBase (CNSA). These datasets were accessed in compliance with the respective database guidelines and regulations for the use of public genomic data.
